# Gut Microbiota-Derived Metabolites in Colorectal Cancer: The Bad and the Challenges

**DOI:** 10.3389/fonc.2021.739648

**Published:** 2021-10-18

**Authors:** Wanru Zhang, Yaping An, Xiali Qin, Xuemei Wu, Xinyu Wang, Huiqin Hou, Xueli Song, Tianyu Liu, Bangmao Wang, Xuan Huang, Hailong Cao

**Affiliations:** ^1^ Department of Gastroenterology and Hepatology, General Hospital, Tianjin Medical University, Tianjin Institute of Digestive Diseases, Tianjin Key Laboratory of Digestive Diseases, Tianjin, China; ^2^ Department of Gastroenterology, The First Affiliated Hospital of Zhejiang Chinese Medical University, Hangzhou, China

**Keywords:** colorectal cancer, gut microbiota, metabolites, tumor immunity, intracellular signal transduction

## Abstract

Accumulating evidence from studies in humans and animal models has elucidated that gut microbiota, acting as a complex ecosystem, contributes critically to colorectal cancer (CRC). The potential mechanisms often reported emphasize the vital role of carcinogenic activities of specific pathogens, but in fact, a series of metabolites produced from exogenous dietary substrates or endogenous host compounds occupy a decisive position similarly. Detrimental gut microbiota-derived metabolites such as trimethylamine-N-oxide, secondary bile acids, hydrogen sulfide and N-nitroso compounds could reconstruct the ecological composition and metabolic activity of intestinal microorganisms and formulate a microenvironment that opens susceptibility to carcinogenic stimuli. They are implicated in the occurrence, progression and metastasis of CRC through different mechanisms, including inducing inflammation and DNA damage, activating tumorigenic signaling pathways and regulating tumor immunity. In this review, we mainly summarized the intimate relationship between detrimental gut microbiota-derived metabolites and CRC, and updated the current knowledge about detrimental metabolites in CRC pathogenesis. Then, multiple interventions targeting these metabolites for CRC management were critically reviewed, including diet modulation, probiotics/prebiotics, fecal microbiota transplantation, as well as more precise measures such as engineered bacteria, phage therapy and chemopreventive drugs. A better understanding of the interplay between detrimental microbial metabolites and CRC would hold great promise against CRC.

## Introduction

Colorectal cancer (CRC) is a leading cause of cancer-related deaths worldwide and the morbidity and mortality of CRC are still rising (1). The mechanism of the initiation and progression of CRC has not been fully elucidated yet, but it is generally believed to be the result of the extensive and complex interaction between genetic and environmental factors. Epidemiological data have demonstrated that adverse environmental exposures, including overweight and obesity, high-fat diet (HFD), smoking and high consumption of alcohol, perform predominant roles in carcinogenesis (2, 3). Significantly, these controllable factors could have a strong impact on the structure and function of gut microbiota.

The microbiota that inhabits the human gut is a key factor in maintaining the stability of the intestinal microecosystem by participating in the immune regulation, substance metabolism, nutrient absorption in the human body, directly or indirectly (4). Usually, the gut microbiome engages in mutualistic relationships with the host, whereas it may mediate the occurrence and development of some diseases, which depends on both environment and the susceptibility of the host. Due to next generation sequencing technologies, such as 16S rRNA, 18S rRNA, internal transcribed spacer (ITS) sequencing, shotgun metagenomic sequencing, metatranscriptomic sequencing and virome sequencing, identifying the characterization of host-microbiota interactions has tremendous progress and the intestinal microbiota is indicated to be closely related to CRC (5, 6). The proposed pathogens which are candidates for CRC mainly include *Fusobacterium nucleatum*, *Escherichia coli, Bacteroides fragilis*, *Streptococcus bovis*, *Enterococcus faecalis* and *Peptostreptococcus anaerobius *(7). Also, the influence of the vaster microbial community, particularly its metabolome, has been connected to CRC (**Table 1**). The gut microbiota, the “new organ” of the human organism, has an enormous metabolic potential (8), and its metabolic capacity greatly exceeds that of human cells (9). About 50% of the metabolites found in feces and urine are derived from, or modified by the gut microbiota (10). Metabolomics, which is the qualitative and quantitative assessment of the metabolites (small molecules<1.5 kDa) in cells, tissues, organs, or biological fluids, has discovered thousands of microbiota-derived metabolites and further expanded our knowledge on the effects of specific metabolites in carcinogenic or cancer-promoting activity (11, 12). Common gut microbiota-derived metabolites mainly include amino acids and their by-products, lipids and lipid-like metabolites, bile acids (BAs) derivatives, and other metabolites produced by the degradation of carnitine and choline (11, 13, 14). These metabolites are altered in CRC patients and some of them have been confirmed to have both local and systemic effects on promoting the risk, initiation and progression of CRC (**Table 2**).

**Table 1:** Summary of individual microbes and their corresponding metabolites probably involved in colorectal cancer.

Microorganism	Biological characters	Metabolites
*Fusobacterium nucleatum*	Gram-negative, anaerobe	Polyamines
		H_2_S
		Fap 2 protein
		Fad A
*Escherichia coli*	Gram-negative, anaerobe	Colibactin
		TMAO
Enterotoxin-producing *Bacteroides fragilis*	Gram-negative, anaerobe	Polyamines
		Bacteroides fragilis toxin
*Enterococcus faecalis*	Gram-positive, facultative anaerobe	Superoxide and hydrogen peroxide
*Peptostreptococcus anaerobius*	Gram-positive, anaerobe	Indole derivatives
*Clostridium*	Gram-positive, anaerobe	DCA
		TMAO
*Desulfovibrios*	Gram-negative, anaerobe	H_2_S
		TMAO

H_2_S, hydrogen sulfide; TMAO, trimethylamine-N-oxide; DCA, deoxycholic acids.

**Table 2:** Summary of studies related to detrimental microbiota-derived metabolites involved in CRC.

Metabolites	Study type	Functions and mechanisms	References
TMAO	Case-control study	The positive association between plasma TMAO and CRC risk	Bae et al. (15)
	Case-control study	TMAO served as a new prognostic marker for CRC	Liu et al. (16)
	Genome-wide systems analysis	Genetically correlation between TMAO and CRC	Xu et al. (17)
DCA	Case-control study	Increased serum DCA levels in patients with colorectal adenomas	Bayerdörffer et al. (18)
			Bayerdörffer et al. (19)
	Prospective cohort analysis	The positive association between fecal DCA and CRC risk	Ocvirk et al. (20)
	Experimental study	Contributing to the development of CRC	Liu et al. (21)
		• Destruction of intestinal barrier function	Dong et al. (22)
		• Inducing intestinal inflammation	Cao et al. (23)
		• Leading to dysbacteriosis	
		• Regulating the monocyte-macrophage system	
	Experimental study	Promoting tumor formation	Cao et al. (23)
		• Inducing RAS–ERK1/2 signaling pathway	Lee et al. (24)
		• Regulating Wnt/β-catenin signaling pathway	Fu et al. (25)
		• Activating PKC/p38 MAPK signaling pathway	Jean-Louis et al. (26)
H_2_S	Case-control study	Sulfidogenic bacteria served as a potential environmental risk factor of CRC	Yazici et al. (27)
	Prospective cohort analysis	The positive association between the sulfur microbial diet and CRC risk	Nguyen et al. (28)
	Experimental study	Inducing DNA damage	Attene-Ramos et al. (29)
		• Genotoxicity mediated by free radical oxygen species	
NOCs	Case-control study	The positive associations between HCAs and the risk of CRC	Zhu et al. (30)
	Experimental study	Increasing DNA damage	Hebels et al. (31)
		• Mutations in K-ras gene with transition G to A	Hebels et al. (32)
		• Inducing oxidative stress	Gottschalg et al. (33)
		• Formation of the NOC-induced DNA adducts	Lewin et al. (34)
HCAs	Case-control study	HCAs exposure as an important pathway for colon carcinogenesis	Helmus et al. (35)
	Prospective cohort analysis	The positive associations between HCAs with the risk of CRC	Cross et al. (36)
	Experimental study	Carcinogenesis and mutagenesis	Hasegawa et al. (37)
		• Inducing DNA damage	
		• Formation of the DNA adducts	
Polyamines	Experimental study	The positive association between plasma polyamine metabolism level and CRC	Liu et al. (38)
			Manna et al. (39)
	Experimental study	Stimulating CRC cells growth	Guo et al. (40)
		Synergistic effect of spermine synthase and MYC	
Ammonia	Case-control study	Contributing to the development of CRC	Clausen et al. (41)
	Experimental study	Promoting neoplastic transformation	Visek et al. (42)
		• Stimulating the growth of cancer cells	
Lactate	Experimental study	Promoting the proliferation, invasion and migration of colon cancer cells	Chen et al. (43)
		• Providing environmental conditions through acidification of TME	Yan et al. (44)
		• Stimulating glycolytic metabolism	
		• Promoting angiogenesis	

CRC, colorectal cancer; DCA, deoxycholic acids; ERK1/2, extracellular-signal regulated kinase 1/2; H_2_S, hydrogen sulfide; HCAs, heterocyclic amines; NOCs, N-nitroso compounds; p38 MAPK, p38 mitogen-activated protein kinase; PKC, protein kinase C; TMAO, trimethylamine-N-oxide; TME, tumor microenvironment.

The current review highlighted the recent advances on the mechanisms by which detrimental metabolites from gut microbiota modulated the development and progression of CRC. Meanwhile, multiple potential therapeutic approaches for CRC were summarized. Targeting small molecule metabolites may contribute to providing promising therapeutic strategies for CRC.

## Interaction of Gut Microbiota, Metabolites, and CRC

The potential genetic changes of CRC have been well confirmed up to now. At least three major molecular pathways can lead to CRC (chromosomal instability pathway, microsatellite instability and CpG island methylation pathway). However, in addition to the existence of several essential mutations, the occurrence and development of CRC also depend on the close interaction of mutagenized cells with the tumor microenvironment (TME) (45). During the development of CRC, tumor-promoting inflammation induced by gut microbiota usually involves the first two, characterized by the exaggerated production of cytokines by resident innate immune cells and the establishment of an immunosuppressive TME. Aberrant inflammation is considered as one of the hallmarks of cancer. During the development of CRC, tumor-promoting inflammation induced by gut microbiota usually involves the first two, characterized by the exaggerated production of cytokines by resident innate immune cells and the establishment of an immunosuppressive TME (45–47). These proinflammatory mediators interact with epithelia to compromise barrier function and further amplify the response by recruiting and activating additional immune cells. The breakdown of the intestinal barrier triggers the invasion of pathogenic microorganisms and their metabolites from the intestinal lumen, and leads to contact between intestinal epithelial cells (IECs) and components of the microbiota that might have pro-tumorigenic characteristics. Ensuring sustained immune response may trigger a cascade of inflammatory changes and promote carcinogenesis (47–49). The causal and complicit roles of microbes in cancer through their effect on the host’s immune system is defined as the immuno-oncology-microbiome (IOM) axis (50).

Pattern recognition receptors (PRRs) are essential components of the host immune system, which can recognize the conserved molecular patterns specific to microorganisms called pathogen-associated molecular patterns (PAMPs) such as lipopolysaccharide (LPS) to trigger diverse innate immune responses (51). The sub-families of PRRs mainly include several types of recognition receptors: the toll-like receptors (TLRs), the NOD-like receptors (NLR), C-type lectin receptors (CLRs), and RIG-I-like receptors (RLRs) (51). Intestinal mucosal epithelial cells and immune cells recognize intestinal microorganisms and their products mainly through TLRs, which are regulated by the intestinal microbiota and by circadian rhythmicity (52–55). In spite of different receptors, the vast majority of TLRs’ signaling is initiated by the myeloid differentiation primary-response protein-88 (MyD88) and leads to activation of downstream signaling pathways and recruitment of transcription factors such as nuclear factor-Kappa B (NF-κB), mitogen-associated protein kinase (MAPK), and interferon (IFN) regulatory factors and subsequent generation of cytokines and chemokines. The expression of TLR2 in colon cancer is significantly upregulated and the TLR2 agonists significantly enhance the proliferation, migration, and invasion of CRC cells (56). TLR4 can produce trophic factors and vascular growth factors through the TLR4/MyD88/NF-κB signaling pathway (57) and promote tumor proliferation through TLR4/Cyclooxygenase 2 (COX2)/prostaglandin E2 (PGE2). TLR9 could induce downstream signals to recruit inflammatory factors, such as IL(interleukin)-8, TGF-β, PGE2, and other immunosuppressive molecules, leading to the continuous state of inflammation, the escape of tumor immunity, and the unlimited proliferation of tumor cells (58).

Immune elimination and immune escape are hallmarks of cancer. The TME is usually at an immunosuppressive state. A range of microbial derivatives and metabolites can mediate host immune disorders *via* affecting the differentiation, proliferation, maturation and effector function of innate and adaptive immune cells, thereby influencing tumor surveillance (47). Polyamine synthesis is essential to induce cytotoxic activity and T-cell proliferation. Supplementation with spermidine or L-arginine promotes homeostatic differentiation of Treg cells with a beneficial role in the context of gut inflammation (59). However, polyamines possibly have opposing roles depending on their concentrations, since elevated polyamine levels in cancer have been shown to diminish the antitumor immune responses by resulting in various defects in immune cell function, including inhibition of lymphocyte proliferation (60–62). Moreover, TME rich in fatty acids can inhibit the function of effector T cells and M1-polarization of macrophages, and facilitate the differentiation of T regulatory cells (Tregs) and M2-like macrophages (63). Notably, only a relatively narrow segment of the studies has elucidated the reciprocal interaction between metabolites and tumor immunity, and their influences on immunosuppression remain poorly characterized.

## Detrimental Microbiota-Derived Metabolites in Intestinal Carcinogenesis

**Figure 1:**
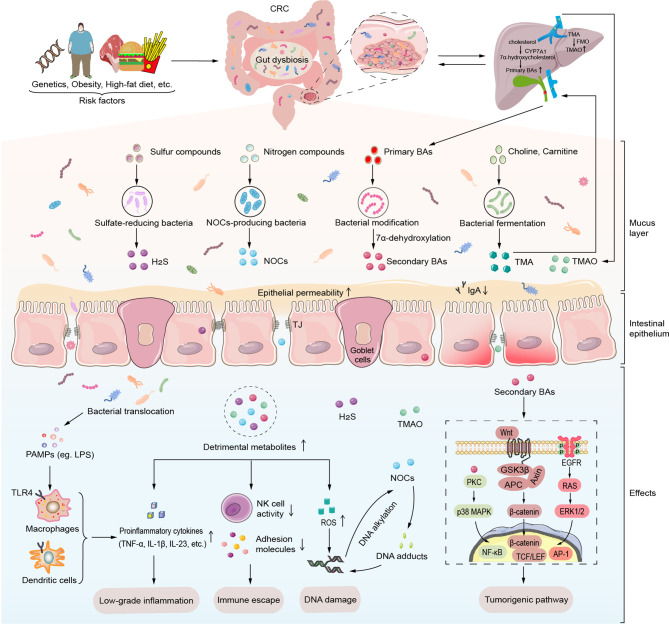
Detrimental gut microbial metabolites induce intestinal carcinogenesis. Various risk factors such as host genetics, obesity, and high-fat diet induce gut microbiota dysbiosis. Alteration of gut microbiota and its metabolites lead to mucus layer thickness reduction, high intestinal permeability and ensuing translocation of commensal microbiota and its metabolites. Maleficent bacteria overgrowth produces amounts of PAMPs like LPS to recognize the TLR4 of macrophages and dendritic cells, which then release certain proinflammatory cytokines (TNF-α, IL-1β, IL-23, etc.). Moreover, detrimental gut microbiota-derived metabolites such as secondary BAs, TMAO, H_2_S, and NOCs induce carcinogenesis through low-grade inflammation, immune escape, DNA damage, and activation of tumorigenic signals. Particularly, secondary BAs contribute to the progression of CRC *via* specific intracellular transduction pathways such as PKC-p38 MAPK signaling pathway, EGFR-ERK1/2 signaling pathway and Wnt/β-catenin signaling pathway. In addition to inducing ROS production, NOCs can involve in DNA damage by DNA alkylation and DNA adducts. AP-1, activator protein-1; APC, adenomatous polyposis coli; BAs, bile acids; CRC, colorectal cancer; CYP7A1, cholesterol 7 α hydroxylase; EGFR, epidermal growth factor receptor; ERK1/2, extracellular signal-regulated kinase 1/2; FMO, flavin monooxygenase; GSK3β, glycogen synthase kinase 3β; H_2_S, hydrogen sulfide; IL-1β, interleukin-1β; IL-23, interleukin-23; LEF, Lymphatic enhancement factor; LPS, lipopolysaccharide; NF-κB, factor-Kappa B; NOCs, N-nitroso compounds; p38 MAPK, p38 mitogen-activated protein kinase; PAMPs, pathogen-associated molecular patterns; PKC, protein kinase C; ROS, reactive oxygen species; TCF, T cell factor; TJ, tight junctions; TLR, Toll-like receptor; TMA, trimethylamine; TMAO, trimethylamine-N-oxide; TNF-α, tumor necrosis factor-α.

One of the primary modes of interaction between the gut microbiota and the CRC is through metabolites. Specific classes of detrimental microbiota-derived metabolites such as Trimethylamine-N-oxide (TMAO), BAs, hydrogen sulfide (H_2_S), N-nitroso compounds (NOCs) form a complex metabolic network related to the etiology and severity of CRC (**Figure 1**). Once these metabolites pass the mucosal barrier, they can act directly on IECs or influence immune responses in the intestinal stroma, trigger the release of pro-inflammatory signals, such as tumor necrosis factor (TNF) and IL-17, or lead to immunosuppression in the TME, which can further promote tumorigenesis. Furthermore, microbial metabolites can induce tumorigenesis by inducing DNA damage and activating the intracellular tumorigenic signaling pathways.

### TMAO and CRC

With the deepening of research on microbial metabolites, TMAO has attracted more attention for its influence on many aspects of health in general. The generation of TMAO is dependent on the gut microbiota, which metabolizes phosphatidylcholine, choline, and carnitine from foods (red meat, egg yolk, milk as typical foods of HFD) to produce trimethylamine (TMA). TMA is carried through the portal circulation to the liver, where it is oxidized by the host hepatic flavin monooxygenase (FMO) to produce TMAO which is then released into circulation and eventually eliminated in the kidney (64–66). Consequently, TMAO is dependent upon dietary consumption of precursors, gut microbial enzymatic activity and host genetics (67), and may be a vital mediator among diet, gut microbiota metabolism and the risk of CRC.

TMAO might be a potential early diagnosis prognostic biomarker for CRC. One study that enrolled 835 matched case-control female pairs from the Women’s Health Initiative Observational Study as research objects showed that TMAO was positively connected with CRC risk in age-adjusted analyses (15). Similarly, Liu et al. found the serum TMAO level of CRC patients was significantly higher than that of healthy controls and was related to tumor progression and distant metastasis (16). In addition, an unbiased data-driven network-based systems approach demonstrated that TMAO was genetically associated with CRC. They found that TMAO might share dozens of genetic pathways including the immune system, cell cycle and Wnt signaling with CRC (17). Although the potential relationship between TMAO and CRC has been investigated, direct evidence for promoting CRC is currently lacking. Inflammation, oxidative stress and DNA damage could be potential molecular mechanisms between TMAO and carcinogenesis. Recently, a dose-response meta-analysis summarized the results of studies on the relationship between circulating TMAO concentrations and inflammation risk, and then revealed a non-linear positive association between increased TMAO concentrations and C-reactive protein (68). Yue et al. demonstrated that TMAO could trigger the activation of the nod-like receptor family pyrin domain containing 3 (NLRP3) inflammasome and inhibit the autophagy gene ATG16L1-induced autophagy to promote the progression of inflammatory bowel disease (IBD) (69). These results implied that inflammation might be a possible factor connecting TMAO and CRC. Oxidative stress could conduce to CRC development by leading tumor cells to be insensitive to antiproliferative signals, apoptosis and anchorage-independent cell growth, and altering the invasion and migration of tumor cells through epigenetic and metabolic mechanisms (70). It has been discovered that increased levels of TMAO in the circulation are related to oxidative stress *via* inducing the production of superoxide, a reactive oxygen species (ROS) (71). Moreover, TMAO involves in the formation of NOCs which cause DNA damage and epigenetic changes, suggesting a potential role of DNA damage in TMAO-contributed carcinogenesis (72). However, the effort of TMAO on the pathogenesis of CRC can be more accurately elucidated through long-term studies of multi-center populations and large samples that include dietary investigation and comprehensive analysis of intestinal target microbial detection as well as combined with animal experiments.

### Secondary BAs and CRC

Primary BAs are synthesized from cholesterol in hepatocytes by cholesterol 7 α hydroxylase (CYP7A1) and released into the intestinal tract with the bile. About 95% of BAs in the intestine are reabsorbed and transported back to the liver *via* the portal. The rest escapes enterohepatic circulation and then enters the colon, where they are transformed by the intestinal bacteria through 7α-dehydroxylation into secondary BAs (73). Bacteria capable of producing secondary bile acids belong to the *B. fragilis*, *Bacteroides vulgatus*, *Clostridium perfringens*, *Eubacterium*, *Lactobacillus* and *Bifidobacterium* (74). The perturbations of the intestinal microbiota composition can strongly impact BA metabolism. It has been reported that interplay between BAs and gut microbiota could mediate the malignant transformation of colorectal adenomas (74), and the elevated levels of secondary BAs, especially deoxycholic acid (DCA) play a critical role in this process. In two small case-control studies from the 1990s, the serum concentration of DCA in colorectal adenoma patients was showed significantly higher compared with healthy individuals (18, 19). Consistent with this, a prospective cohort analysis investigated the association between gut microbial co-metabolism and the risk of CRC in Alaska Native and rural African people. Data manifested that fecal concentrations of the DCA were more than 2-fold higher in Alaska Native than that in rural African participants (20). Several experimental findings supported these clinical data. Our group has unveiled partial mechanisms of DCA promoting the pathogenesis of CRC using a mouse model of gastrointestinal tumorigenesis. Data showed that DCA brought about an increase in the number and volume of intestinal adenomas in *Apc^min/+^* mice, resulting in impaired intestinal barrier function and intestinal inflammation, and subsequently promoted intestinal carcinogenesis *via* activating tumor-related signaling pathways. (21–23).

In addition, some related tumorigenic signaling pathways by which DCA promotes the development of CRC have been identified and studied intensively (73). First of all, DCA triggered tyrosine phosphorylation and activated the epidermal growth factor receptor (EGFR) signaling pathway of tyrosine kinases in a ligand-dependent manner, which then led to the activation of RAS-extracellular-signal regulated kinase 1/2 (ERK1/2) signaling. ERK1/2 could induce the activation of activator protein 1 (AP-1) to mediate cell proliferation and differentiation (24). Moreover, DCA-induced Wnt signaling is able to induce an inflammatory response and stimulate the proliferation of CRC cells, which also plays a crucial role in the progression of CRC (23, 25). DCA can affect the development of cancer by causing β-catenin to be released and enter the cytoplasm where it translocates to the nucleus and stimulates transcription factors such as the T cell factor/lymphoid enhancer factor (TCF/LEF) family (75). Besides, perturbation of the plasma membrane by DCA also activates protein kinase C (PKC). PKC subsequently induces the activation of p38 mitogen-activated protein kinase (p38 MAPK) and NF-κB, which play indispensable roles in the regulation of immune and inflammatory responses. Activated NF-κB translocates into the nucleus, where it contributes to the expression of IL-1β (26, 76).

BAs receptors also play critical roles in CRC. Farnesoid X receptor (FXR) and Takeda G protein-coupled receptor 5 (TGR5) are the two most critical receptors of BAs, which mediate the regulation of BAs on downstream signaling pathways and cell functions (74). The regulation of the microbiota-FXR axis is one of the main mechanisms that gut microbiota affects and participates in the metabolism of BAs. Colonization of GF mice with a human microbiota from feces can reduce total BA levels and induce expression of FXR and its downstream target genes in the intestine (77). Currently, it is generally accepted that the deletion and decrease of FXR expression in the colon and rectum are associated with CRC development and metastasis (78, 79). FU et al. observed that down-regulation of FXR promoted the progression of CRC, whereas selective activation of intestinal FXR could restrict intestinal cancer stem cell proliferation and profoundly increased survival in *APC^min/+^* mouse models of adenoma (25). Moreover, a study has already found that the TGR5 mRNA expression level in intestinal tumor tissues was significantly higher than that in normal tissues (80). Nevertheless, the role of TGR5 in intestinal carcinogenesis remains to be explored.

### H_2_S and CRC

H_2_S produced by fermenting different S-containing substrates notably cysteine through both the gut microbiota and endogenous enzymes is emerging as a key regulator of gut health, including CRC and IBD (81, 82). Gut luminal H_2_S production appears to be dependent on the action of sulfate-reducing bacteria (SRB). Therefore, the upregulation of H_2_S and sulfidogenic bacteria may be potential environmental risk factors contributing to CRC development (82).

Previous studies have shown that African Americans contain substantially higher abundances of SRB and *Bilophila wadsworthia* in the colon, which may be the reason why the incidence of CRC in African Americans is higher than in non-Hispanic whites (27). In particular, the abundance of sulfur-metabolizing bacteria correlated positively with fat and protein intake. Long-term persistence to a dietary pattern related to sulfidogenic bacteria in stool can cause an elevated risk of distant CRC (28). These data support the conception that H_2_S participates in colorectal carcinogenesis. The important carcinogenic mechanism may be the cytotoxicity and genotoxicity of H_2_S. H_2_S has been reported to induce genomic DNA damage that could be radical-mediated, which is directly related to the induction of cumulative mutations of CRC (29). In addition, H_2_S is cytotoxic to intestinal epithelium cells and causes mucosal damage (83, 84). In addition, H_2_S is cytotoxic to intestinal epithelium cells and causes mucosal damage (29, 83). An increase in SRB and H_2_S concentration could reduce disulfide bonds in the mucus network, thereby allowing luminal bacteria and their products to penetrate and contact with the host epithelial lining and immune cells to induce apoptosis in epithelial cells and inflammatory activation in immune cells (85, 86). What’s intriguing is that there indeed exists conflicting data on the protective and pathological effects of H_2_S in the gastrointestinal tract. It has been argued that H_2_S can control the inflammation and facilitate correction of microbiota biofilm dysbiosis and mucus layer reconstitution (87, 88). Also, H_2_S could inhibit proliferation and promote protective autophagy in colon epithelial cells *via* the AMPK pathway (89). These controversies may relate to the bell-shaped dose-response curve of H_2_S. Overall, further studies are needed to determine how H_2_S produced by the gut microbiota contributes to CRC pathogenesis.

### NOCs and CRC

NOCs, including N-nitrosamines and N-nitrosamides, are among the most potent experimental procarcinogens mainly derived from the fermentation of proteins of red and processed meat by facultative and anaerobic colonic bacteria (90, 91). Types of dietary intake and bacterial colonization largely affect the formation of NOCs in the intestine. The high levels of red meat consumption would increase the total amount of NOCs and consequently account for the epidemiologic association between red meat consumption and CRC (92). In addition, advances from clinical studies further substantiated the hypothesis that NOCs intake might be associated with a higher CRC incidence in humans (30).

N-nitrosamines are pro-carcinogens and have no direct mutagenic effect on the cells of organs and tissues. However, cytochrome P450 family 2 subfamily E member 1 (CYP2E1)-mediated hydroxylation of N-nitrosamines can result in the formation of carcinogenic surfactant diazomethane and ultimately lead to the generation of DNA-reactive methyl carbocation (93). N-nitrosamides are direct carcinogens that can interact with the cellular macromolecule. The mechanism of NOCs inducing carcinogenesis is known to relate to DNA damage. On the one hand, NOCs are very likely to react with the nucleophilic center of DNA bases, causing DNA alkylation and inducing the K-ras gene and TP53 gene in epithelial cells to undergo G→A transitions (31, 94). Similarly, oxidative damage is another important form of DNA damage caused by nitrosamine exposure (32). On the other hand, the generation of extremely reactive alkylating agents like diazoacetate contributes to the formation of the NOC-induced DNA adducts which are the main executor of DNA-damaging and carcinogenic properties (33, 34). The imbalance between DNA damage and DNA repair determines the initiation and progression of CRC to some extent. By way of example, if not or incorrectly repaired by O(6)-Methylguanine-DNA-methyltransferase (MGMT), the NOC-specific DNA adduct O6-carboxymethyl-2′-deoxy-guanosine (O6-CMdG) would cause dramatic biological consequences, such as accumulating on the DNA of intestinal cells, promoting the shedding of colon cells, and leading to the early stage of CRC (95, 96). Therefore, how to reduce or eliminate the content of NOCs may provide a good prospect for the prevention of CRC.

### Other Metabolites and CRC

A diverse reservoir of metabolites from gut microbiota includes the well-known substances mentioned above, the more underestimated examples such as heterocyclic amines (HCAs), polyamine, ammonia and lactate, which also participate in the initiation and promotion of CRC.

Another important metabolite is HCAs, which can be significantly produced by gut microbiota during fermentation of red meat and processed meat. Evidence is available that individual differences in the gut microbiota have a strong impact on the genotoxic and carcinogenic properties of HCAs. *Bacteroides* strains tend to help convert HCAs into DNA-reactive carcinogens, whereas probiotic *lactobacilli* binding HCAs can reduce their mutagenic effect (97, 98). Numerous studies have implicated HCAs as an oncogenic driver in CRC, due to the association between elevated HCAs levels and high risk of CRC (35, 36). HCAs have been reported to induce frameshift mutations, microsatellite instability, strand breaks and oxidative base damage (99). Particularly, HCA-induced DNA adducts which are considered as the main executor of DNA-damaging and carcinogenic properties can generate mutations in the colon (37).

Most tumors have a greatly increased need for polyamines to meet their huge metabolic demands. Intracellular polyamine levels are maintained through tightly regulated pathways of biosynthesis, decomposition, absorption, and output. Polyamines and their metabolites in urine and plasma are often regarded as possible biomarkers of occurrence and progression in CRC (12). A piece of strong evidence is that the levels of putrescine and agmatine were described to increase with the progression of CRC (38, 39). Additionally, Guo et al. found the synergistic effect of spermine synthase and oncogene MYC increased polyamine metabolites, suggesting dysregulation of polyamine metabolism has been linked to the development of CRC (40).

According to reports, ammonia is one of the potential carcinogenic products of protein fermentation. Amino acid deamination by the gut bacteria and urea hydrolysis by bacterial urease are the main sources of ammonia in the intestine (100, 101). Continuous exposure of colonocytes to free ammonia may contribute to the development of CRC (41). The concentration of ammonia in the human intestinal cavity progressively increases from the right colon to the left colon, with the highest levels in the region of the colon where cell proliferation and the incidence of polyps and cancer are the highest (102). Animal experiments showed that after long-term ammonium chloride perfusion into the colon of rats, the distal colon mucosa displayed histological damages with the epithelial tissue disordered and the pathologic intestinal epithelial shedding (103). Besides, excessive ammonia enhances cell proliferation in the colon mucosa, which makes neoplastic transformation more efficient (42).

In tumors, both hypoxia and oncogene expression can stimulate glycolytic metabolism. As a result, the production of lactate seems to be a common energy metabolism pathway in cancer cells (104). Lactate can stimulate angiogenesis and transfer nutrients such as oxygen and glucose to cancer cells, and consequently inducing colon cell proliferation, invasion and migration (43, 44). Furthermore, the presence of lactate in the TME leads to extreme acidic conditions, inhibits the cytotoxic and effector functions of T cells and advances an immune escape of CRC, and even hampers the efficacy of several chemotherapeutic agents resulting in a poor prognosis (105, 106).

## Clinical Transformation of the Gut Metabolome in CRC

Metabolomics has been increasingly used to identify biomarkers in disease with great potential for clinical translation. With relevant technological advances and new analytical and bioinformatics tools development, metabolomics can already provide sensitive and highly reproducible platforms allowing the quantification of the known compound or comprehensive analysis of all the measurable analytes in a sample (107). A large amount of metabolomics data has been accumulated to be used to investigate the interaction between the host and microorganisms from the perspective of metabolism. It was found that several gut microbiome-associated serum metabolites (GMSM) have changed significantly through integrated analysis of the serum metabolites and fecal metagenomics of patients with CRC and adenoma, which can efficiently discriminate patients with CRC and adenoma from normal individuals (108). Dysregulation of these metabolites indicates the possibility of common diagnostic biomarkers for CRC. However, there is still a need for better non-invasive biomarkers for CRC, especially for the early stages of the disease, including the adenoma step and the initial CRC stages. The content of several classes of bioactive lipids, including polyunsaturated fatty acids, secondary BAs and sphingolipids (109, 110) increased in adenoma patients. Most of these metabolites show directionally consistent changes in patients with CRC, indicating that these changes may represent early events of carcinogenesis (111). Rao J et al. pointed out that three metabolites (hydroquinone, leucenol and sphingomyelin) are positively and significantly correlated with CEA and/or CA 19-9, which may be potential biomarkers for advanced CRC (112). Furthermore, dynamic shifts of microbial metabolism have been confirmed in patients with different stages of colorectal neoplasia. Yachida et al. showed that branched-chain amino acids and phenylalanine were significantly increased in patients with intramucosal carcinomas, and BAs were significantly elevated both in patients with multiple polypoid adenomas or intramucosal carcinomas (113). These data provided evidence for the functional importance of the gut metabolome in CRC and implied a potential role of the gut microbiota-derived metabolites in the early diagnosis and prognosis of CRC. The term “pathogenic function (pathofunction)” has been proposed to evaluate specific features of host bacterial communities based on the detrimental metabolites produced by the gut microbiota. The pathofunction of the gut microbiota may contribute to the development of precision treatment directing gut microbiota to increase host health. However, universal biomarkers for CRC detection have not been identified due to the high variability of the microbiota and their metabolites between individuals. A standardized sample preparation and metabolomics analysis methods warrant further exploration.

## Manipulation of Metabolites in CRC Treatment

In view of the importance of the mechanistic link between detrimental gut microbiota-derived metabolites and CRC, targeted metabolomics has been a remarkable research topic in recent years. There have been several beneficial metabolites [i.e., short-chain fatty acids (SCFAs)] profiling studies for preventing and treating CRC, however, fewer studies have been involved in CRC therapeutic explorations by targeting detrimental microbial-derived metabolites. In our review, we put effort to mention those studies which have linked regulating excess detrimental microbial-derived metabolites in the diagnosis, prevention and treatment of CRC. Pay more attention to how to use these studies for clinical application has great significance for the diagnosis, prognosis, therapeutics, and prevention of CRC.

## Diet Modulation

Diet is an important environmental determinant of metabolism. Diet-mediated changes in whole-body metabolism and systemic nutrient availability can affect the overall performance of TME (2, 114). Metabolites derived from gut microbes are the key executors of diet affecting the host physiology, either inhibiting or accelerating tumor growth. Red and processed meat might increase CRC risk by increasing the formation of detrimental microbial metabolites such as TMAO, NOCs and HCAs. On the contrary, dietary fiber (DF) enriched in fruits, vegetables and whole grains is associated with a lower risk of CRC (115). A comprehensive meta-analysis of prospective studies and clinical trials provided convincing evidence that fiber intake was related to the risk of CRC. Dose-response relationships suggested greater effects on CRC risk reduction for higher intakes of DF (116). Importantly, DF can protect against CRC by altering not only microbial composition but also the concentration and availability of metabolites. The link between DF and BAs may be identified as a potential mechanism to explain the protective role of DF in CRC. In a comparative study in African American individuals, switching from low-fiber, high-fat to high-fiber, low-fat diet resulted in significant improvement in the microbiota and metabolome associated with CRC risk. Further exploration revealed that it was mainly due to an increase of saccharolytic fermentation and butyrogenesis and inhibition of secondary BAs synthesis (117). Consistent with this, a recent study investigated concentrations of fecal and serum BAs in vegans and omnivores, showing a vegan diet low in fat and high in fiber was positively correlated with fecal BA concentrations (118). Additionally, Li et al. have suggested that supplying the DF diet to mice can reduce TMA and TMAO outputs *via* inhibiting intestinal microbial TMA lyase activity (119). Thus, DF intervention for individuals with various clinical statuses is expected to become an emerging treatment against CRC.

## Probiotics/Prebiotics

Probiotics are well-known biologically active candidates for the treatment of several diseases and unhealthy conditions by positively regulating the gut microbiota (120). *Lactobacilli*, *Clostridium butyricum* and *Lactobacillus rhamnosus GG* (LGG) are some of the most studied and well-characterized among probiotics. On the one hand, probiotics are commensal live bacteria that reduce intestinal inflammation and improve intestinal barrier function to achieve the prevention and treatment of CRC. On the other hand, probiotics can remove carcinogenic metabolites and toxins from the gastrointestinal tract, compete with spoilage bacteria and pathogenic bacteria and maintain the balance of gut microbiota (121, 122). Detrimental metabolites are mainly derived from the gut microbiota, such as *Bacteroides*, *Clostridium* (clusters XIVa and XI), *Eubacterium*, *Enterobacter, Campylobacter jejuni* and SRB. Supplementing probiotics can reduce the colonization of these bacteria, which may reduce the level of detrimental metabolites in the intestine or body, and thus inhibit intestinal tumor development. Probiotics can regulate intestinal bacteria related to proteolysis, reduce harmful protein fermentation, thus reducing the toxicity of metabolites (123). It was found that supplementing with *Lactobacillus* or *Bifidobacterium* or both can significantly reduce serum cholesterol. The main mechanism is the use of bile salt hydrolyzing enzymes to decouple the bile, and then precipitation of cholesterol along with the decoupled bile (124, 125). Moreover, Our previous studies have found that *Clostridium butyricum* antagonized against HFD-induced intestinal carcinogenesis and decreased the production of secondary BAs *via* down-regulating the abundance of *Clostridium* (126).

Fructooligosaccharides and galactooligosaccharides and inulin are frequently reported prebiotics. Prebiotics are non-digestible selectively fermented DF that can be used as substrates by gut microbiota to promote the metabolism of lipids, proteins and minerals, and produce metabolites that are potentially protective of gut functionality (127). In addition, prebiotics promote favorable bacteria of the indigenous intestinal flora of humans, or also improve the survival of probiotics that have been ingested at the same time, thereby indirectly reducing potentially pathogenic bacteria and/or detrimental metabolites in the intestine. Interestingly, the introduction of prebiotics improves the viability of probiotics. Compared with used alone, the combination of prebiotics and probiotics has been reported to reduce the expression of genes involved in carcinogenic pathways and drug resistance, and decrease the levels of the metabolite lactate, thus disturbing the growth of cancer cells and benefiting for CRC treatment (128). However, clinical trials evaluating the efficacy of probiotics for CRC are still limited and there are no specific criteria for selecting probiotics or prebiotics, thus systematic evaluation of different strains is required.

## Fecal Microbiota Transplantation

Fecal microbial transplantation (FMT) is an emerging treatment scheme for transplanting gut microbiota from healthy donors to patients through various channels, so as to restore the intestinal microbial diversity of patients and achieve the therapeutic purpose (129). The significant differences in gut microbiota composition between cancer patients and healthy individuals show the therapeutic potentials of microbiota modulation. It is reasonable to support the hypothesis that FMT is prospective in cancer management as well as cancer-treatment associated complications, which is being actively explored (130, 131). The natural gut microbiome could promote host fitness, eliminate excessive inflammation and protect against mutagen/inflammation-induced colorectal tumorigenesis (132). FMT affects metabolism by resolving dysbiosis, or directly transferring primary and secondary BAs, which may underlie the observed effects of FMT in CRC. At this stage, the uncertainty and safety of the efficacy of FMT still should not be ignored. In-depth experiments and clinical trials are essential for its development and improvement.

## Engineered Bacteria

Therapeutic approaches that use live tumor-targeting bacteria are ushering in a new era of cancer therapy. Bacteria can be engineered to act as therapeutic delivery payloads following simple genetic rules or complex synthetic bioengineering principles to realize the functions of delivering drug molecule or immunoregulatory factors to tumors, destroying tumor matrix, and silencing tumor genes (133). Compared with non-precision approaches such as the use of probiotics, engineered bacteria can be more accurately mitigate the effects of detrimental microbial-derived metabolites that are significant in the pathogenesis of CRC. A recent study developed engineered *Escherichia colistrain* that could convert systemic ammonia into L-arginine in a mouse model, thus exhibiting the potential for microbiota-based therapeutics to more precisely regulate metabolism (134). Engineered bacteria can be applied either as a monotherapy or a complement to other anticancer therapies to obtain improved antitumor activities. Some of them have passed clinical tests and shown greatly encouraging results. In fact, the success of therapy depends on the functional stability, clinical potency and safety of the engineered bacteria (135). Meanwhile, we must identify appropriate gut-adapted strains and consider performance metrics when deploying such bacteria *in vivo* (136).

## Phage Therapy

Phage therapy may be a promising therapeutic tool for modulating the gut microbiota and gut metabolome as well as stimulating systemic anticancer immune response (137). Bacteriophages are viruses ubiquitous in the human intestinal tract that infect and replicate within bacteria and usually have species-level specificity. By directly knockdowning their bacterial targets or further inducing cascading effects on non-susceptible species through inter-bacterial interactions, the therapeutic effect of bacteriophages can be utilized to modulate the gut microbiota, which in turn has a potential impact on gut metabolome such as significant changes in bile salts (138, 139). The link between phage and microbial metabolites provides an interesting therapeutic avenue. Manipulation of microbiota by lytic phage can be used to selectively reduce detrimental microbial metabolites (140). For instance, phage predation of *E. faecalis* reduced tyramine, which can induce ileal contractions (141).

## Chemopreventive Drugs

The value of chemoprevention strategies for preventing early stages or recurrence of CRC or new polyp formation has been widely noted in recent decades. Several studies to date have supported the chemopreventive potency of some promising agents, particularly those targeting metabolic pathways. Inhibition of the formation of detrimental metabolites appears, therefore, to be a rational target in chemoprevention.

Ursodeoxycholic acid (UDCA) is one of the BAs that has different effects on CRC compared with DCA. A population-based study indicated that the use of UDCA was associated with a lower risk of CRC (142). UDCA treatment affected the microbial community composition in men, as manifested by increased abundance of *Faecalibacterium prausnitzii* and decreased *Ruminococcus gnavus*, which partially explained the UDCA action to inhibit adenoma development (143). Additionally, the hydrophobicity of BAs may be an important determinant of their carcinogenic properties (144). One possible biologic mechanism by which UDCA acts therapeutically may be to increase the hydrophilicity of the biliary pool, dilute the concentration of toxic secondary BAs in the biliary pool such as DCA and lithocholic acid (LCA) and then prevent colonic neoplastic transformation (145). However, only low-dose UDCA is recommended for the prevention of colorectal neoplasia. The hypothesis that UDCA may increase the risk of developing neoplasia was first proposed by Eaton et al., who showed that high-dose UDCA can increase the incidence of colorectal neoplasia in patients with ulcerative colitis and primary sclerosing cholangitis (146). Obviously, more studies are needed for UDCA applications.

CRC and diabetes mellitus share various clinical risk factors leading to an interest in anti-diabetic agents as potential chemopreventive drugs for CRC. Metformin, as the first-line oral medicine for type 2 diabetes, has been shown to significantly reduce colorectal adenoma and cancer incidence and improving survival outcomes (147, 148). In the context of CRC, the protective effects of metformin are likely multi-factorial. One of the antitumor effects of metformin is an indirect effect resulting from systemic metabolic changes, including decreases in plasma glucose. Metformin had strong effects on the gut microbiome, and this was reversed when the drug was removed (149). Exposure to metformin modulated gut microbiota in patients with type 2 diabetes and increased the concentration of BA glycoursodeoxycholic acid and tauroursodeoxycholic acid, which were known antagonists of the FXR. These changes inhibited intestinal FXR signaling and improved metabolic dysfunction, hinting the alterations in microbiome composition and the subsequent impact on secondary BAs production may be an important factor in the prevention of CRC by metformin (150). The other is a direct effect on tumor cells. The liver kinase B1 (LKB1)-dependent activation of AMPK and reduction of mammalian target of rapamycin (mTOR) activity may be significant contributors to the inhibitory effects of metformin on cancer cell growth and proliferation (151).

The polyamine metabolic pathway is a potential target for cancer chemoprevention. A polyamine-blocking therapy (PBT) by targeting their synthesis and transport can exert antitumor effects. It not only altered the levels of polyamines in many tumors, but also relieves the immunosuppression in the TME, characterized by an increase in granzyme B+, IFN-γ+ CD8+ T-cells, and decreased immunosuppressive cell levels (60, 152). Ornithine decarboxylase (ODC) and S-adenosylmethionine decarboxylase (AdoMetDC), as the rate-limiting enzyme of polyamine biosynthesis, are the key factors in the regulation of polyamine levels. Difluoromethylornithine (DFMO) is an irreversible inhibitor of ODC and recognized as a chemopreventive and chemotherapeutic agent to decreases cancer hallmarks including enhanced cell proliferation and apoptosis resistance (153). To date, several phase II and Phase II studies on DFMO in the treatment of CRC have been reported. DFMO may prevent CRC by reversing Ca^2+^ channel remodeling, activating a synergistic re-expression of aberrantly silenced tumor-suppressor genes and modulating DNA hypomethylation (154, 155). To inhibit polyamine synthesis, many AdoMetDC inhibitors have been discovered from the first-generation inhibitor methylglyoxal bis (MGBG) to the third-generation inhibitor AbeAdo and tested in clinical trials treating cancers (156). However, none of them has been finally approved for clinical use due to low efficiency or strong side effects. In addition, given that the polyamine transport system is upregulated in cancers, a range of polyamine analogs and polyamine-like structures have been synthesized. They can interrupt polyamine biosynthesis and compete for uptake, and thus reduce the normal polyamine content required for cell growth (157).

## Conclusion and Perspectives

A convergence of basic research, epidemiological and clinical studies is illuminating the contribution of detrimental gut microbiota-derived metabolites to the initiation and progression of CRC. The microbial metabolites described above, specifically TMAO, BAs, H_2_S and NOCs, work as critical signaling molecules that mediate crosstalk between the microbes and host, and play pivotal roles in colorectal carcinogenesis. They represent potential biomarkers for the early diagnosis and prognosis of CRC.

Although it is known that the detrimental gut microbiota metabolites contribute to intestinal malignant lesions in numerous ways, most of them have not yet been functionally characterized. In particular, the in-depth molecular mechanisms involved in the interaction between metabolites and CRC, as well as direct synergy between metabolites remain to be elucidated. Of note, there are still controversial findings that parts of the detrimental metabolites exhibit both the properties of anti-carcinogenic and pro-tumorigenic, which may depend on many factors, such as their luminal concentrations, the duration of the colonic stasis, interactions with other metabolites and tumor developmental stage. However, based on large-scale epidemiological studies and clinical outcome trials, we still believe that targeted regulation of detrimental metabolites to eliminate or reduce their concentration is expected to be effective strategies for the prevention and treatment of CRC. Furthermore, more studies are necessary to ensure the functional stability, clinical potency and safety of management measures.

## Author Contributions

WZ, YA, and XQ were the major contributors to the writing and revision of the manuscript. XMW, XYW, and HH performed the literature search and data analysis. XS and TL critically revised the manuscript. BW supervised the entire project. XH and HC were involved in the study design and the critical review and revision of the manuscript. All authors contributed to the article and approved the submitted version.

## Funding

This work was supported by the grants from the National Natural Science Foundation of China (82070545 and 81970477) and the Key Project of Science and Technology Pillar Program of Tianjin (20YFZCSY00020).

## Conflict of Interest

The authors declare that the research was conducted in the absence of any commercial or financial relationships that could be construed as a potential conflict of interest.

## Publisher’s Note

All claims expressed in this article are solely those of the authors and do not necessarily represent those of their affiliated organizations, or those of the publisher, the editors and the reviewers. Any product that may be evaluated in this article, or claim that may be made by its manufacturer, is not guaranteed or endorsed by the publisher.

## Abbreviations

BAs, bile acids; CRC, colorectal cancer; DCA, deoxycholic acid; DF, dietary fiber; DFMO, difluoromethylornithine; FMT, fecal microbiota transplantation; FXR, farnesoid X receptor; H_2_S, hydrogen sulfide; HCAs, heterocyclic amines; HFD, high-fat diet; IL, interleukin; LPS, lipopolysaccharide; MAPK, mitogen-associated protein kinase; NF-κB, nuclear factor-Kappa B; NOCs, N-nitroso compounds; PAMPs, pathogen-associated molecular patterns; PRRs, pattern recognition receptors; ROS, reactive oxygen species; SRB, sulfate-reducing bacteria; TGR5, Takeda G protein-coupled receptor 5; TLRs, Toll-like receptors; TMA, trimethylamine; TMAO, trimethylamine-N-oxide; TME, tumor microenvironment; UDCA, ursodeoxycholic acid.
